# Psychometric evaluation of three-factor eating questionnaire -R18 in aging Finnish men with increased risk for type 2 diabetes

**DOI:** 10.1177/02601060221112178

**Published:** 2022-07-11

**Authors:** Katriina Malkki-Keinänen, Maria Lankinen, Leila Karhunen, Ursula Schwab

**Affiliations:** 1 School of Medicine, Institute of Public Health and Clinical Nutrition, 4344 University of Eastern Finland, Finland; 2 Department of Medicine, Endocrinology and Clinical Nutrition, 60650 Kuopio University Hospital, Finland

**Keywords:** Eating behaviour patterns, aging men, TFEQ-R18, factor analysis

## Abstract

**Background:**

Deeper comprehension of eating-related behaviour (how and why people eat) can reveal new aspects to support health and prevent type 2 diabetes (T2D). However, such research is largely missing in aging men.

**Aim:**

The aim was to investigate suitability of the Three-Factor Eating Questionnaire-R18 (TFEQ-R18) in Finnish aging men which is widely used to examine factors: cognitive restraint (CR), uncontrolled eating (UE), and emotional eating (EE).

**Methods:**

Study population consisted of 420 men aged 50–75, who completed the TFEQ-R18 at the baseline of the T2D-GENE lifestyle intervention study. Inclusion criteria were impaired fasting glucose (IFG) and body mass index ≥25 kg/m^2^. Confirmatory factor analysis was used to study psychometrics (reliability, validity, and model fit) and factor structure of TFEQ-R18.

**Results:**

The items loaded to the three factors (CR, UE, EE) as in previous studies, except two items at CR factor and one at UE factor, which were therefore omitted. UE was also discovered split into two sub factors (named as ‘craving’ and ‘loss-of-control’), UE being a higher-order (h) factor. The resultant revised version was named as Three-Factor Eating Questionnaire Revised to 15-items with higher-order factor (TFEQ-R15h).

**Conclusion:**

The original 18-item version of the TFEQ was not optimal in the population consisting of Finnish aging men with elevated T2D risk. A modified 15-item version of the TFEQ could be used to describe EB in this population instead.

## Introduction

More knowledge is needed about why and how people eat and choose their food. Eating behaviour (EB) is the key for understanding people's food choices (Bellisle, 2003). Although EB has already been studied for decades there is still surprisingly little research on EB of aging men. There are some suggestions that as men age, they become less susceptible to external incentives of food and emotion-induced eating (Harden et al., 2009). As external and emotional eating are EB features which have been associated with risk of obesity and weight gain (Burton et al., 2007; James et al., 2017), diminishing the features should ease to stop weight gain (Coffino et al., 2018; Michels et al., 2020). Yet, excess weight is substantially common in European aging men (Corona et al., 2010) despite that the increase of body mass index (BMI) clearly lowers life expectancy (Robertson et al., 2013). Men's waistline increase (Han et al., 2017) which along with overweight and obesity (Robertson et al., 2013) are independent risk factors to various non-communicable diseases such as cardiovascular disease (CVD) and type 2 diabetes (T2D) (Finelli et al., 2013). Obese people also have a higher risk for COVID-19 and higher mortality rate (Popkin et al., 2020). The prevalence of T2D increasing in all age groups causes costs to society as well as complications to individuals (Zheng et al., 2018). It is estimated that almost 200 million people aged 65 or over will have T2D by 2030 (Sinclair et al., 2020). However, there is substantial evidence that the risk of T2D and CVD can be lowered by lifestyle, which includes healthy diet and decrease of body weight (Merlotti et al., 2014; Tuomilehto et al., 2001). Adherence to a healthy diet also improves cognition and decreases frailty of old age (Casas et al., 2022).

EB features have also been linked to several health outcomes. Ability to restrain one's eating has been associated with healthy food choices (Brogan and Hevey, 2013; de Lauzon et al., 2004) and ability to maintain weigh loss (Keränen et al., 2009). High scores in Uncontrolled eating (UE) are associated with overeating and obesity (Keränen et al., 2009; Provencher et al., 2003; Vainik et al., 2019) whereas eating due to external incentives has relationship with impulsivity (Kakoschke et al., 2015), less healthy food choices (Brogan and Hevey, 2013), and high total energy intake (Paans et al., 2019). In depression, external eating mediates higher intake of fast food (Paans et al., 2019), while emotional eating plays a role of consumption of sweet foods (Konttinen et al., 2010). In addition, overeating may occur if there is insulin resistance in the brain due elevation of disinhibition (Kullmann et al., 2015). Therefore, better understanding of EB could reveal important aspects influencing health in aging men.

EB is usually measured by self-reported questionnaires. The most commonly used EB questionnaire, called Three-Factor Eating Questionnaire (TFEQ) or Eating Inventory, consists of 51 items (Stunkard and Messick, 1985). In the development of the questionnaire, three factors describing distinctive features of EB were found: Cognitive restraint, Disinhibition, and Hunger.

Later, Karlsson et al. (2000) evaluated the scaling properties and construct validity of the TFEQ with over 4000 obese middle aged men and women (Karlsson et al., 2000). As a result, construct validity was supported for three factors: Cognitive restraint (CR), while majority of items assigned to Hunger and Disinhibition formed a factor which was labelled as UE. The third factor contained items related to overeating during dysphoric mood states and was labelled as Emotional eating (EE). The revised version consisted of 18 items and was accordingly named TFEQ-R18 (Karlsson et al., 2000).

Factor structure, construct validity, and reliability of TFEQ-R18 have been investigated in many populations, including young women (Anglé et al., 2009), undergraduate students (Martins et al., 2020), overweight adults (Chong et al., 2016), depressed adults (Paans et al., 2018), and have been compared between normal- and overweight women (Brytek-Matera et al., 2017), and between teens and middle-aged adults (de Lauzon et al., 2004). Despite some low item-factor loadings, several researchers (Anglé et al., 2009; Brytek-Matera et al., 2017; Chong et al., 2016; de Lauzon et al., 2004; Martins et al., 2020; Paans et al., 2018) suggest maintaining the three-factor structure. Yet, some have made modifications to factor variables of the TFEQ-R18 due to lack of validity or reliability (Halali et al., 2020; Kavazidou et al., 2012; Mostafavi et al., 2017; Pentikäinen et al., 2018) and in one study UE was split into two subfactors (Yabsley et al., 2019).

Individual items of the questionnaire have revealed that reliability of CR factor is most compromised. Several studies with normal and overweight, young and middle-aged adults (Bond et al., 2001; Bryant et al., 2018; Cappelleri et al., 2009; Chong et al., 2016; Halali et al., 2020; Julien Sweerts et al., 2019; Kavazidou et al., 2012; Mostafavi et al., 2017; Pentikäinen et al., 2018; Szakály et al., 2020) and in children (Yabsley et al., 2019) have discovered that especially items 15, 16 and 18 from CR factor load poorly at the TFEQ-R18 version. In addition, 14 from UE factor loaded poorly at TFEQ-R18 with obese north American adult population (Cappelleri et al., 2009; Szakály et al., 2020). It has been suggested that these variables are not well suited to all cultural settings (Cappelleri et al., 2009). In addition, UE factor has been found to split into internal and external hunger (Yabsley et al., 2019) much as the Hunger factor in the 51-item version (Bond et al., 2001). (See description of items in Supplementary material S1).

Furthermore, many behavioural aspects of eating may not be generalisable from women to men, due to gender-specific physiological features. Such are hormonal (i.e. cortisol and progesterone have increasing effect on food intake and increase dysregulated eating, whereas testosterone has reversed effect) and neural systems (i.e. obese women have altered reactivity in reward systems in response to stress) (Anversa et al., 2021). Men have generally more external eating and crave for different foods than women (Burton et al., 2007). Women have more commonly eating disorders (Anversa et al., 2021) as well as loss of control -type of eating (Anversa et al., 2021; Strand et al., 2021). In addition, women tend to diet more often than men (Emery et al., 2021; Halali et al., 2020), possibly since they are more susceptible to social pressure (Shatenstein et al., 2016) and social rejection (Anversa et al., 2021) also in romantic relationships (Côté and Bégin, 2020) than men, although there are some culture-specific differences in attitudes toward body shape (Sicilia et al., 2020). In addition, associations of personality traits with body weight are mediated differently between men and women (Sutin and Terracciano, 2017).

Accordingly, TFEQ scores differ between men and women so that women typically have higher scores of UE and EE than men (Cappelleri et al., 2009; de Lauzon-Guillain et al., 2006; Stinson et al., 2019; Svensson et al., 2014). However, most studies have not differentiated the factor scores between genders (Karlsson et al., 2000; Kavazidou et al., 2012; Löffler et al., 2015; Szakály et al., 2020), which also points the need of further research on the differences of EB between men and women.

Therefore, because EB as well as the functionality of TFEQ-R18 as a measure of EB are still poorly understood among aging men with elevated T2D risk, the aim of the current study was to investigate the psychometric properties of the TFEQ-R18, specifically in this population.

## Methods

### Participants

This is a cross-sectional analysis from the baseline of the lifestyle intervention T2D-GENE-study (T2D-GENE study). Altogether 635 Caucasian men aged 50 to 75 were recruited to the study from Northern Savonia, Finland (Schwab et al., 2021), among those men who had participated in the METabolic Syndrome In Men (METSIM) study previously (Stančáková et al., 2009). Inclusion criteria of the T2D-GENE study were impaired fasting glucose (IFG) (fasting plasma glucose 5.6–6.9 mmol/l) with (2-h glucose 7.8–11.0 mmol/l) or without impaired glucose tolerance (IGT) (2-h glucose <7.8 mmol/l), HbA1c <6.5%, BMI ≥25 kg/m^2^ and belonging to lowest or highest tertile of the genetic risk score for T2D (low risk or high risk, respectively) (Stančáková et al., 2017). Men with type 1 or 2 diabetes or isolated IGT (fasting plasma glucose <5.6 mmol/l and 2-h plasma glucose 7.8–11.0 mmol/l), HbA1c ≥6.5%, or other chronic diseases, which prevented participation, were excluded from the T2D-GENE study.

At the baseline of the T2D-GENE study, 420 participants filled in the Three-Factor Eating Questionnaire revised 18-item version (TFEQ-R18) and were included in this study. Subjects baseline characteristics of the current study are presented in Table 1. The 215 men who did not fill in the questionnaire were younger (mean 64.2  ±  5.8 years) than those who filled in the TFEQ-R18 (p  =  0.001).

**Table 1.** Baseline characteristics (n  =  420).

Baseline characteristics	Mean ± SD / n (%)
Age (y)	66 ± 6
Body mass index (kg/m^2^)	28.7 ± 3.1
Waist circumference (cm)	104 ± 9
Fasting plasma glucose (mmol/l)	6.0 ± 0.3
120 min plasma glucose (mmol/l)	6.3 ± 1.6
HbA1c (mmol/mol)	37 ± 3
Education^a^	
primary school	70 (17)
secondary level	242 (60)
high	78 (19)
other	16 (4)
Living^b^	
with a partner	363 (87)
alone	53 (13)
Food management responsibility^c^	
with a partner	336 (81)
alone	51 (12)
not at all	30 (7)

a : n  =  406, ^b^: n  =  416, ^c^: n  =  417, y: years, HbA1c: Glycated haemoglobin.

The protocol for this study was approved by the Ethical committee of the hospital district of Northern Savo. Participants signed the informed consent statement.

### Eating behaviour

The Three-Factor Eating Questionnaire (TFEQ-R18) (Karlsson et al. 2000) translated to Finnish was used to assess EB. The questionnaire measures distinctive features of EB by three factors. The factor Cognitive restraint (CR) contains six items, Uncontrolled eating (UE) nine items, and Emotional Eating (EE) three items. The items 1 through 17 were responded on a 4-point Likert scale and item 18 on an 8-point scale, which was converted to 4-point scale. Items 1–13 were reverse scored as recommended (de Lauzon et al., 2004). There were no missing items since the questionnaire was filled at website which could only be saved after each variable had a reply. However, some men filled the questionnaire on paper and brought it to the first group meeting. The questionnaire was checked at return and in case of missing values, participants were asked to re-fill them. The items of TFEQ-R18 are presented in Supplement S1 (note that Finnish translation of items 14 and 17 somewhat differ from those of the original English ones).

Factor scores were rescaled to 0–100% [((raw score – lowest possible raw score)/possible raw score range)*100] for compatibility reasons (de Lauzon et al., 2004). Higher percentage conveys that person has higher tendency towards the feature measured by the factor.

### Laboratory measurements

Baseline laboratory visit included 2-h oral glucose tolerance test (OGTT) along with measurements of height, weight, waist circumference, and body composition. The measurements were performed after 12 h of fasting by trained nurses. The methodology has been previously described (Stančáková et al., 2009).

### Statistical analysis

IBM SPSS (version 27) was used for data analysis. The psychometric properties of the TFEQ-R18 three-factor model were analysed with Confirmatory factor analysis (CFA) with AMOS 27. The data were screened for missing values before any further tests or factor analysis. Skewness and kurtosis of variables (acceptable if between −2 and + 2) as well as whether the eighteen variables and the three assumed factors correlate significantly. This was done by bivariate correlation with Spearman correlation coefficients and two-tailed significance.

With CFA reliability, construct validity, model fit, and method bias were tested with Maximum likelihood (Hu and Bentler, 1999). Path diagram and standardized estimate loadings as well as error variance of items were checked as they indicate how well each item represents the factor (Hair et al., 2014b). Ideal for standardized estimate loading is 0.700 or above but as low as 0.500 is acceptable. However, these are ‘rules of thumb’, not absolute values indicating a variable must be deleted (Hair et al., 2014b).

Internal reliability scale of factors in CFA was estimated with Composite Reliability (CompR) which must be above 0.700 for each separate factor (Hair et al., 2017). In addition to CompR, also Cronbach's Alfa was used as another indicator of internal reliability (Cronbach, 1951; Gliem and Gliem, 2003; Trobia, 2008). Level ≥0.700 was accepted if there were 4 or more items, and if a factor consisted two or three items, cut-off point ≥0.600 was considered acceptable (Multon and Coleman, 2010). Reliability of factors was assessed for each factor separately.

Internal validity of the construct was tested by estimating the factor correlations and Average variance extracted (AVE) (Fornell and Larcker, 1981; Hair et al., 2014a; Lowry and Gaskin, 2014). The square root of AVE has to be above the factor correlations to indicate appropriate variance (Hair et al., 2017; Henseler et al., 2015). Maximum shared variance (MSV) was checked since discriminant validity is acquired when AVE is higher than MSV (Hair et al., 2014a; Henseler et al., 2015). The squared multiple correlation less than 0.20 was considered as indication of high level of error (Hooper et al., 2008). If discriminant validity posed an issue, we were to examine possible higher-order (also called second-order) factors with some distinct subfactors (also called first-order factors) (Byrne and Stewart, 2006; Iacobucci, 2010). However, TFEQ-R18 is known to have issues with discriminant validity between Uncontrolled Eating and Emotional Eating factor in line them being under same construct in the original Eating Inventory (Bond et al., 2001; Stunkard and Messick, 1985).

The convergent validity of construct measuring difference between factors was examined with both AVE (strict measure) or Composite Reliability (CompR; lenient measure). Cut-off point for AVE was 0.500 and 0.700 for CompR (Hair et al., 2017). However, AVE with slightly lower threshold than 0.500 was accepted if there were no problems with discriminant validity (if MSV was lower than AVE) (Wright et al., 2012). Validity was checked from the model with a free tool (Gaskin et al., 2019).

Model fit included commonly used indices: relative chi-square (X^2^/df), incremental and goodness-of-fit index (Comparative fit index; CFI) and two absolute fit indices Root mean square error of approximation, (RMSEA) and Standardised root mean square residual (SRMR) (Hair et al., 2017). As there are no absolute indices but rather ranges of fit index values which are investigated in context with the underlying theory, model fit was considered as good when: X^2^/df between 1.000–3.000, CFI >0.950, SRMR <0.080, RMSEA <0.060 (Hooper et al., 2008; Hu and Bentler, 1999; Iacobucci, 2010). These were chosen since chi-square estimates how well the data fits the overall model, whereas CFI takes sample size into account, SRMR informs the difference between the sample and hypothesised model, and RMSEA considers residual variance of the model (Hooper et al., 2008). Model fit measures were checked from the model and calculated with a free tool (Gaskin and Lim, 2016).

Distinguishability of the factors at the questionnaire was examined with method bias tests. Method bias was assessed by two methods: correlation of sub factors and multicollinearity. If the correlations of sub factors were below 0.900, the method bias was not extreme (Lowry and Gaskin, 2014; Paans et al., 2018).

In addition, Spearman correlation coefficients was used to analyse the associations between TFEQ factors and baseline characteristics (age, BMI, waist circumference) (Table 2).

**Table 2.** Associations of factor scores in TFEQ-R18 and TFEQ-R15h with baseline characteristics (n  =  420).

			Age	BMI	Waist (cm)
TFEQ-R18	**CR** **6 items**	r	0.055	0.013	0.018
	**UE** **9 items**	r	−0.147**	0.206***	0.228***
	**EE** **3 items**	r	−0.096*	0.158**	0.144**
TFEQ-R15h	**CR** **4 items**	r	0.040	0.030	0.038
	**UE high** **8 items**	r	−0.136**	0.204***	0.224***
	**UE1** **craving** **3 items**	r	−0.081	0.146**	0.164**
	**UE2** **loss of control** **5 items**	r	−0.152**	0.204***	0.223***
	**EE** **3 items**	r	−0.096*	0.158**	0.144**

**BMI: body mass index kg/m^2^, CR**: Cognitive Restraint, **UE**: Uncontrolled Eating, **EE**: Emotional Eating, **UE high**: higher-order factor of Uncontrolled Eating, **craving**: subfactor 1 of Uncontrolled Eating, **loss of control**: subfactor 2 of Uncontrolled Eating, **r**: Spearman's Correlation Coefficient, * p < 0.05, ** p < 0.01, *** p < 0.001.

## Results

### Descriptives of TFEQ-R18

Means and standard deviations of TFEQ-R18 factors were the following: CR 49.8%  ±  15.6, UE 27.3%  ±  15.8, and EE 18.7%  ±  20.4. Ranges of factors were CR 0–100%, UE 0–92%, EE 0–100%.

### Preliminary examination of data

Skewness and kurtosis were at acceptable level. Skewness was between −0.553 and 1.093 (with standard error of 0.119), and kurtosis between −0.792 and 0.527 (with standard error of 0.238). Items of CR were most negatively skewed, and all items of EE were above 1.

Items at UE and EE factors correlated well within the factors. Items at CR correlated mostly within the factor, but item 16 “How likely are you to consciously eat less than you want?”, did not correlate with items 12 “I do not eat some foods because they make me fat”, and 15 “How frequently do you avoid stocking up on tempting foods?” of CR. Factors CR and UE, as well as UE an EE were discovered to correlate with each other, but CR did not correlate with EE (Supplementary table S2).

### Testing TFEQ with CFA

The R18 version had acceptable reliability for UE and EE but CR had low values (Table 3). Reliability of factors was assessed with Cronbach's alfa which indicated poor fit for CR at R18 version. Discriminant validity was acceptable only for EE (AVE 0.610). Fit indices were at acceptable level with R18 version although goodness-of-fit index was low (CFI 0.920).

**Table 3.** Reliability, validity, and model fit indices of Confirmatory factor analysis (n  =  420).

		TFEQ-R18	TFEQ-R15h	Threshold or cut-off point
Deleted items		-	14, 15, 16	
CompR	CR	0.685	0.716	0.700 at least
	UE	0.818	0.823	
	EE	0.823	0.900	
AVE	CR	0.292	0.398	0.500 at least
	UE	0.338	0.610	
	EE	0.610	0.819	
MSV	CR	0.016	0.008	less than AVE
	UE	0.383	0.465	
	EE	0.383	0.465	
square root	CR	0.541	0.631	> than factor correlation
	UE	0.582	0.781	
	EE	0.781	0.905	
α	CR	0.663	0.700	0.700 at least
	UE	0.816	0.812	
	EE	0.816	0.816	
X^2^/df		2.183	1.874	1-3
CFI		0.920	0.959	>0.950
SRMR		0.069	0.059	<0.080
RMSEA		0.053	0.046	<0.060

**TFEQ-R18**: Three-Factor Eating Questionnaire-R18 (Karlsson et al. 2000), **TFEQ**-**R15h**: the version which has 15 items and high-order factor of UE, **Threshold**: cut-off point or goal, **Deleted items**: item numbers of TFEQ which were deleted from revision, **CompR**: Composite reliability, **AVE**: Average Variance Extracted, **MSV**: Maximum Shared Variance, **square root**: of AVE, **α**: Cronbach's alfa, **CR**: Cognitive Restraint, **UE**: Uncontrolled Eating, **EE**: Emotional Eating, **X2/df**: relative Chi-square, **CFI**: Comparative fit index, **SRMR**: Standardized root mean square residual, **RMSEA**: Root mean square error of approximation.

Standardised residual values improved from 6.921 (between EE and UE in R18-version) to 1.426 (between EE and CR in R15h-version) and were no longer significantly associated at the R15h version whereas they were associated in other versions.

Reliability was compromised with the TFEQ-R18 version, and validity was low which means that all items are not measuring what they are assumed to measure (Gliem and Gliem, 2003; Henseler et al., 2015). Item 16 (CR) was omitted due reliability issues; however convergent validity was yet compromised. Low AVE for CR and UE was partially sorted by deleting items 14 (UE) and 15 (CR). However, discriminant validity of UE was still inadequate since correlation of factors UE and EE was too high. AVE was less than MSV and AVE for UE was low. In addition, square root of AVE for UE was less than its correlation with EE. Therefore, other options were explored for.

The best construct validity with adequate reliability and good fit indices were at TFEQ-R15 version which had two sub factors under the higher-order UE (Table 3). Thus, was ended up with the 15-item version of TFEQ (items 14, 15, and 16 deleted) with UE factor split in two subfactors which are under a higher-order factor. It was named accordingly TFEQ-R15h. At this R15h version factor CR includes four items and higher-order UE eight items. EE remained as it was at R18 version. Higher-order UE was split into UE1 and UE2 which were renamed describing the item themes. UE1 was called craving (includes items 1, 5, and 7): items describe craving food *before* eating. The UE2 was called loss of control (items 4, 8, 9, 13, and 17): items identify difficulty of eating cessation *while* eating.

**Figure 1.**
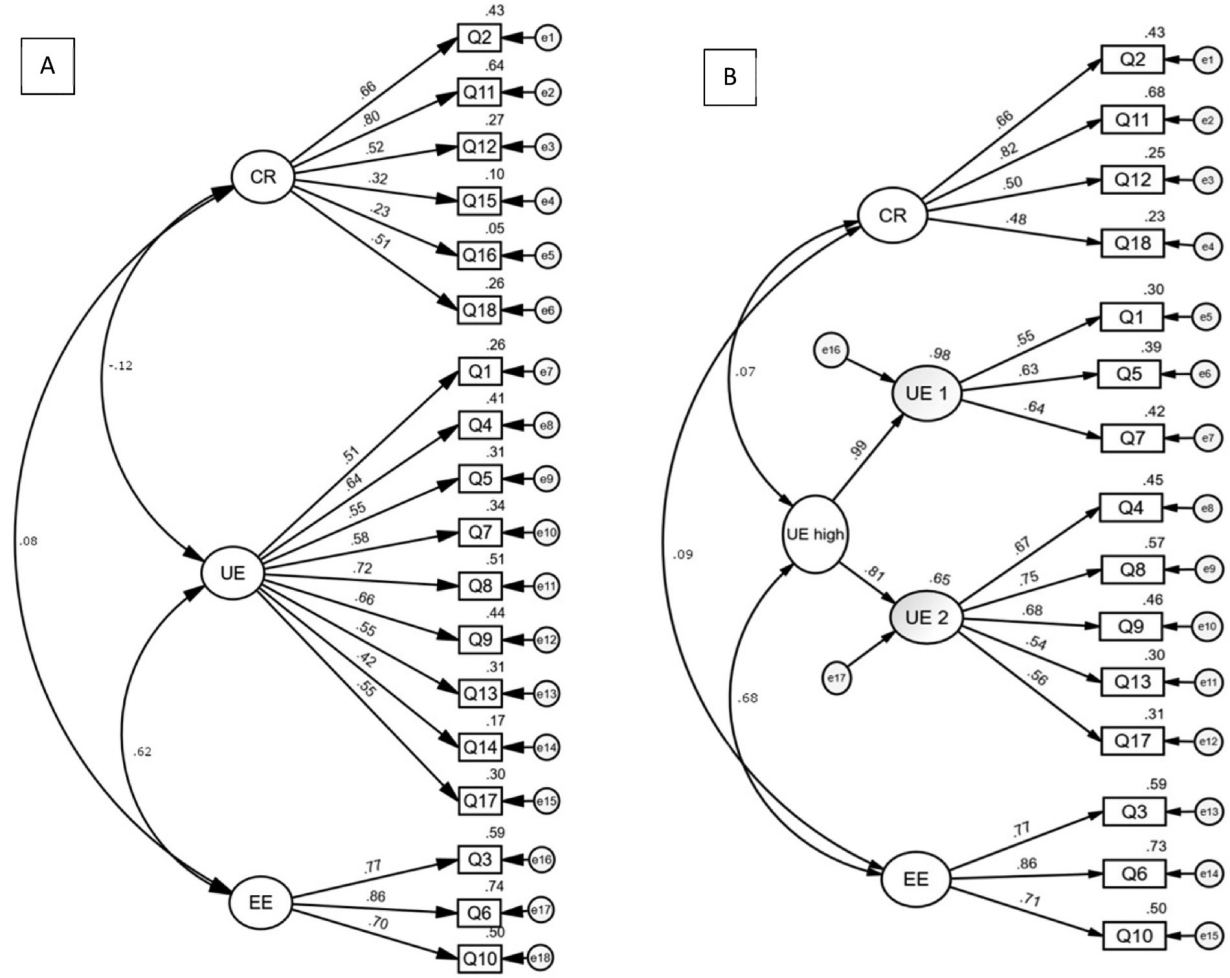
Path diagrams of confirmatory factor analysis (CFA) of TFEQ-R18 (a) and TFEQ-R15h (B). View of standardized estimate loadings. CFA was conducted to measure strengths of different features of EB: Cognitive restraint (CR), uncontrolled eating (UE), and emotional eating (EE). Boxes labelled Q1-Q18 are items of the questionnaire, circled e1-e18 are error variance.

Path diagrams of TFEQ-R18 and TFEQ-R15h are shown in Figure 1. Reliability, validity, and model fit indices of Confirmatory factor analysis of TFEQ from 18- and 15-item versions are shown at Table 3.

Reliability with Cronbach's alfa for UE1 and UE2 were adequate (0.633 and 0.772, respectively). Method bias was also assessed by correlation of subfactors of the updated version. All the factors of TFEQ-R15h were below 0.900 indicating distinct difference.

Four-item CR correlated negatively with UE2 (loss of control) (Supplementary table S2). UE1 (craving) and UE2 correlated with each other. EE correlated significantly with UE1 and UE2.

The TFEQ-R15h version indicated thus best validity, reliability, and good fit indices. It also showed some improvement at method bias correlation test

### Descriptives of TFEQ-R15h

In TFEQ-R15h, the mean and standard deviation of CR was 50.5  ±  18.6%, UE 27.5  ±  16.6%, and sub-factors of UE: UE1 (craving) 26.1  ±  17.0% and UE2 (loss of control) 24.8  ±  17.2% (raw scores of CR and UE were rescaled to match the original ratio with adjusted formula, see Methods). Ranges of factors were CR: 0–100% and UE: 0–91.7%, UE 1: 0–81.2%, UE 2: 85.9%. The descriptives of EE remained the same as in TFEQ-R18.

Correlations of baseline characteristics with factor scores of TFEQ-R18 and TFEQ-R15h are presented in Table 2. CR of R18 or R15h versions was not associated with any of the measured characteristics. UE and EE of both TFEQ versions had similar associations with BMI as well as waist circumference the associations being stronger with UE. In addition, both UE and EE factors were negatively associated with age, and also here the stronger association with UE was shown. The subfactor UE2 (loss of control) had remarkably similar associations as UE high. Associations of UE1 (cravings) with baseline characteristics were in line with UE high with BMI and waist circumference whereas age did not correlate. Fasting and 120-min plasma glucose as well as Glycated haemoglobin (HbA1c) were checked but there were no associations with any of the factors (data not shown).

Factor correlations of both versions are seen in Figure 1. At R18 version CR and EE had no significant association but CR and UE were negatively (p < 0.100), and UE and EE positively associated (p < 0.001). At R15h version only UE and EE had significant positive correlations (p < 0.010).

## Discussion

The aim was to assess the psychometric properties of the TFEQ-R18 in aging males with an increased risk for T2D to obtain more information about functionality of TFEQ-R18 as a measure of EB in this cohort. The construct reliability and validity of the TFEQ-R18 was compromised and results support the use of the 15-item version of TFEQ with higher-order factor (TFEQ-R15h) in this population.

Our results were consistent with previous research since the same items (i.e. items 14 “How often do you feel hungry?”, 15 “How frequently do you avoid stocking up on tempting foods?”, and 16 “How likely are you to consciously eat less than you want?” from factors UE, CR and CR of R18, respectively), have been found challenging also in many other studies using TFEQ-R18 (Bond et al., 2001; Bryant et al., 2018; Cappelleri et al., 2009; Chong et al., 2016; Halali et al., 2020; Kavazidou et al., 2012; Mostafavi et al., 2017; Pentikäinen et al., 2018) or the questionnaire using same items (Yabsley et al., 2019). For example, factor structure indicated low loadings for items 15 (stocking up) of CR and items 14 (hunger frequency) as well as items 1 “When I smell a sizzling steak or see a juicy piece of meat, I find it very difficult to keep from eating, even if I have just finished a meal” and 17 “Do you go on eating binges though you are not hungry?” of UE in the obese Asian cohort (Chong et al., 2016). In addition, low standardized regression coefficient loading estimates were seen to items 1 (sizzling steak) and 14 (hunger frequency) (UE) in a Brazilian validation study with young adults (Martins et al., 2020), and analysis of TFEQ-R21 in obese adults in northern America by Cappelleri also indicated low estimate to item 14 (hunger frequency) (2009). On the other hand, the same item loaded to two factors (UE and EE) amongst overweight women with mean age over 60 but not amongst men of same age and weight group at the study (Cornelis et al., 2014). Furthermore, low communalities were shown in a Finnish study with young females to items 4 (UE) and 15 (stocking up) and 16 (consciously eat less) (CR) (Anglé et al., 2009). These items have thus shown implications of poor loadings toward their primary factor, even in different kind of populations. The way how different items and their response options are formulated could provide an explanation. Statement-type items from 1 to 13 are all similarly scaled on four options. From item 14 onward there are question-type claims with varying response options. Even though it is recommended that type of answering varies within a questionnaire (Podsakoff et al., 2012), varying response types may not be the best option for some responders as Podsakoff and colleagues also state. For instance, Mazzeo et al. changed response-type in some items to a 4-point Likert-scale and removed the items in which the Likert-scale was not optimal (alike items 14–18 at 18-item version) (Mazzeo et al., 2003).

Additionally, items 14 and 15 include frequency qualifiers which are described as indeterminate words (such as ‘sometimes’ or ‘almost always’) (Podsakoff et al., 2012). Furthermore, item 15 deals with two deep structures about availability of tempting foods: does a person have foods that lure her/him, and how often temptations are avoided (Johnson, 2004). Similar double structures are found at item 16, too. It includes a presumption that a person would wish to eat less than he/she prefers to eat. In addition, the item inquires how likely this predetermined behaviour is to happen even if the respondent has not attempted suppress eating. The omitted items may thus have seemed ambiguous to respondents. Moreover, item 14 was translated in Finnish version “How often do you desire food?” which is not accurately same as it is in the original TFEQ-R18 (“How often do you feel hungry?”) (Karlsson et al., 2000). It is therefore possible that these discrepancies have also contributed to the inconsistency of the responses in this item.

The discovery of the correlation tests was that in the 18-item version CR and EE were uncorrelated while CR and UE were slightly correlated. However, discriminant validity challenge with the factors was no longer present after omitting item 14. UE and EE were not discriminant neither in R18 or R15h versions, but they have been found to have a common ground also previously (Bond et al., 2001). This is likely due to the fact that the three EE items were included in Disinhibition construct of the original 51-item questionnaire (Bond et al., 2001; Karlsson et al., 2000; Stunkard and Messick, 1985; Westenhoefer et al., 1999).

Improvement was noticed of model fit indices from R18 to R15h version. Surprisingly, also reliability and construct validity of EE improved from R18 to R15h version although items of EE were unaltered. This is likely due to improvement of UE features at R15h version. R15h version had low method bias detected by variance inflation factor. In addition, the subfactors UE1 ‘craving’ and UE2 ‘loss of control’ found in this study, were distinct, and no major multicollinearity was found. Improvement of fit measures CFI and X^2^/df indicate that omitting the three items (14, 15, 16) which correlated with more than one factor and a discovery of higher-order factor, clarified the overall questionnaire. The updated version was thus more precise for this cohort.

CR has been found to split into two (Westenhoefer et al., 1999) or three (Bond et al., 2001) sub factors. However, our study did not support the distinction of restraint factor into subfactors as R18 includes only few items from original Flexible and Rigid control subfactors, i.e. items 2 “I deliberately take small helpings as a means of controlling my weight” and 11 “I consciously hold back at meals in order not to gain weight” of Flexible control and one item (15; stocking up) of rigid control (Westenhoefer et al., 1999). Instead of finding subfactors, the items 15 and 16 were omitted due to vast shared variance with UE factor.

Finding of the current study of UE split to two subfactors is interestingly comparable with a finding from Yabsley and colleagues who found four factors while validating the children's version of TFEQ-R21 (Yabsley et al., 2019). The current craving subfactor (items 1 (sizzling steak), 5 “Being with someone who is eating often makes me hungry enough to eat also”, 7 “When I see a real delicacy, I often get so hungry that I have to eat right away”) was identical with their External UE factor. In addition, our loss of control subfactor (items 4 “Sometimes when I start eating, I just can’t seem to stop”, 8 “I get so hungry that my stomach often seems like a bottomless pit”, 9 “I am always hungry so it is hard for me to stop eating before I finish the food on my plate”, 13 “I am always hungry enough to eat at any time”, 17 (eating binges)) was almost identical with their Internal UE factor, with the exception that the study of children had good loading also for hunger frequency item corresponding item 14 at R18. Moreover, items 8 (bottomless pit) and 13 (always hungry) (part of loss of control subfactor) were equivalent to items 24 and 34 that formed a part of ‘internal locus of hunger’ -subfactor in TFEQ-51 (Bond et al., 2001). Similarly, items 5 (hungry when others eat) and 7 (eat delicacy right away) in R18, part of Craving subfactor, were equivalent to item 19 and 22 that formed external locus of hunger scale in TFEQ-51 (Bond et al., 2001). Therefore, it is possible that the factor structure of split UE could display those two features of EB, i.e., whether people have external craving of food or internal feeling of loss of control while eating.

Moreover, as disinhibition has been reported to be elevated in obese men regardless of age (Harden et al., 2009), it would be essential to understand the reasons, pathways and consequences of high disinhibition and hunger scores. Increased craving (externality) has been associated i.e. with fast-food intake, which in turn has been associated with increased BMI (Burton et al., 2007). Craving is also associated with binge eating in overweight adults (Chao et al., 2016). Frequency of food craving is associated with external and emotional eating (Coffino et al., 2018). The loss of control -type (internal) eating is associated with greater alcohol intake in young adult men (Brosof et al., 2019). On the other hand, Niemeier et al. found that eating in regard of internal UE is associated with weight regain in middle-aged people whereas external UE was not (Niemeier et al., 2007). Homeostatic (physiological) desire of food could play a role between the differences whether people gain weight or not (Burton et al., 2007). There is a need to distinguish constructs within TFEQ at varying populations (Bond et al., 2001; Julien Sweerts et al., 2019; Westenhoefer et al., 1999). Therefore, more specific factors of UE locus are justified and portray complexity of EB.

Men in our study had similar UE and EE scores as a population consisting middle-aged French men (de Lauzon et al., 2004) or Finnish middle-aged men with average BMI 26.7 kg/m^2^ (Konttinen et al., 2010). These results support the hypothesis that middle-aged or older men have generally lower UE and EE than women and young (Anversa et al., 2021; de Lauzon et al., 2004). In line with this was also the finding of significant negative correlation between age and UE or EE in the present study. Intriguingly, only the subfactor loss of control of UE had significant negative correlation with age whereas the subfactor craving did not. This suggests that the observed association between age and UE was related to internal rather than external control of eating.

BMI correlated with UE and EE. Positive association between BMI and EE has been found in earlier studies, for instance in an adult cohort (de Lauzon-Guillain et al., 2017). UE has correlated positively with BMI in young Finnish females (Anglé et al., 2009) as well as in gender mixed adult cohorts from USA (mean BMI 27.2 kg/m^2^) (Verzijl et al., 2018) and Germany (Löffler et al., 2015). Also CR has been found to have positive correlation with BMI in young and normal weight adults but not in obese subjects (Bryant et al., 2018; de Lauzon-Guillain et al., 2006), CR and BMI correlate also in nonobese women but not in obese men (Provencher et al., 2003). Thus, in line with earlier findings in obese men there was no significant correlation between CR and BMI either in the present study. Löffler et al. discovered that in a German sample of middle-aged adults the relationship of CR and BMI had a reversed U-shape association (BMI was high if a person had medium CR). All three factors were associated positively with BMI also when controlled for age, gender and education, but UE had the strongest association with BMI among these three factors (Löffler et al., 2015). It also needs to be noted that even if high CR is found in normal-weight adult subjects, high CR is not premonition for weight gain in these subjects (de Lauzon-Guillain et al., 2006). Some subgroups have also shown conflicting results since an association with CR and BMI was found in obese adults without diabetes whereas there were no association in obese adults with diabetes or non-obese adults without diabetes in a North-American cohort (Cappelleri et al., 2009).

There are divergent associations with eating patterns between sexes and many behavioural aspects of eating are not generalizable from women to men (Anversa et al., 2021; Brosof et al., 2019; Burton et al., 2007; Opwis et al., 2017). Food intake is regulated by the brain, an organ which is affected for instance by insulin (Heni et al., 2015) and other hormones (Asarian and Geary, 2013). Women have fluctuation of hormones and a significant change of hormonal levels when they age (Hirschberg, 2012) which are different from men. Therefore, the brain driven appetite might not be entirely equal in men and women and could cause altered responses to questions related to EB. In addition, other behavioural differences between sexes comprise e.g. loss of control sensations in men (Strand et al., 2021), brooding about eating in women (Opwis et al., 2017), and personality features such as perseverance in women and extraversion in men are differently associated with BMI (Oniszczenko and Stanisławiak, 2019). These behavioural features have quite different outcomes with eating styles and may affect BMI through differing underlying causes between sexes. Therefore, EB would be justified to be examined separately in men and women.

The strength of the study is that it was a relatively large-scale study investigating EB among previously largely unexplored group, aging men with increased risk for T2D. The study also highlights the need for examining EB separately in men, in addition to cohorts of different ages and body weight. Moreover, since our study consisted of a sample of homogenous participants it enabled us to assess psychometric attributes of the TFEQ-R18 translated to Finnish and provide an applicable version of the original measure.

It is a limitation that only 420 men of 635 participants recruited answered TFEQ-R18 at baseline. The men who did not reply were about 2 years younger than those who filled out the questionnaire. In general, the participants of the current study were probably more motivated and interested in diet, health and related factors than their peers in general population since all participants had participated earlier in the METSIM study (Stančáková et al., 2017), potentially affecting the generalisation of the present findings. Although the R15h version of TFEQ was shown to be a feasible method to study EB in aging men with elevated risk of T2D, it should be assessed also in other groups, including aging women as well as healthy aging men to understand its functionality in diverse groups.

## Conclusions

In summary, this study suggests that the revised 15-item, higher order version of the TFEQ could be used to study EB among aging men with elevated risk for T2D. The results highlight also internal and external dimensions of uncontrolled eating to be identified in this particular group. More research is, however, needed to confirm the findings of the present study.

## Supplemental Material

sj-docx-1-nah-10.1177_02601060221112178 - Supplemental material for Psychometric evaluation of three-factor eating questionnaire -R18 in aging Finnish men with increased risk for type 2 diabetes Supplemental material, sj-docx-1-nah-10.1177_02601060221112178 for Psychometric evaluation of three-factor eating questionnaire -R18 in aging Finnish men with increased risk for type 2 diabetes by Katriina Malkki-Keinänen, Maria Lankinen, Leila Karhunen and Ursula Schwab in Nutrition and Health

sj-docx-2-nah-10.1177_02601060221112178 - Supplemental material for Psychometric evaluation of three-factor eating questionnaire -R18 in aging Finnish men with increased risk for type 2 diabetes Supplemental material, sj-docx-2-nah-10.1177_02601060221112178 for Psychometric evaluation of three-factor eating questionnaire -R18 in aging Finnish men with increased risk for type 2 diabetes by Katriina Malkki-Keinänen, Maria Lankinen, Leila Karhunen and Ursula Schwab in Nutrition and Health

## Abbreviations

EB: eating behaviour
TFEQ-R18: Three-factor Eating questionnaire, revised 18-item version
CR: cognitive restraint
UE: uncontrolled eating
EE: emotional eating
UE1: Craving -type of UE
UE2: Loss of control -type of UE
TFEQ-R15h: Three-factor Eating questionnaire, revised 15-item version
T2D: type 2 diabetes
CVD: cardiovascular disease
FR: food record
BMI: Body mass index (kg/m^2^)
HbA1c: glycated haemoglobin
IFG: impaired fasting glucose
IGT: impaired glucose tolerance
OGTT: oral glucose tolerance test
CFA: Confirmatory factor analysis
X^2^/df: relative chi-square
CFI: Comparative fit index
RMSEA: Root mean square error of approximation
SRMR: Standardised root mean square residual
AVE: Average variance extracted
MSV: maximum shared variance
CompR: composite reliability
