# Supplementary material for: Psychometric evaluation of three-factor eating questionnaire -R18 in aging Finnish men with increased risk for type 2 diabetes
Source: Nutr Health. 2022 Jul 11;30(2):279–90. doi: 10.1177/02601060221112178 (PMC11141102; doi:10.1177/02601060221112178)
Supplement: sj-docx-1-nah-10.1177_02601060221112178 - Supplemental material for Psychometric evaluation of three-factor eating questionnaire -R18 in aging Finnish men with increased risk for type 2 diabetes [file sj-docx-1-nah-10.1177_02601060221112178.docx]

Psychometric evaluation of Three-Factor Eating Questionnaire -R18 in ageing Finnish men with increased risk for type 2 diabetes

**Supplementary material S1.** Item content* of TFEQ-R18 scales and the Finnish questionnaire

1. When I smell a sizzling steak or delicious food, I find it very difficult to keep from eating, even if I have just finished a meal. (UE)^1^
2. I deliberately take small helpings as a means of controlling my weight. (CR)
3. When I feel anxious, I find myself eating. (EE)
4. Sometimes when I start eating, I just can't seem to stop. (UE)^2^
5. Being with someone who is eating often makes me hungry enough to eat also. (UE)^1^
6. When I feel blue, I often overeat. (EE)
7. When I see a real delicacy, I often get so hungry that I have to eat right away. (UE)^1^
8. I get so hungry that my stomach often seems like a bottomless pit. (UE)^2^
9. I am always hungry, so it is hard for me to stop eating before I finish the food on my plate. (UE)^2^
10. When I feel lonely, I console myself by eating. (EE)
11. I consciously hold back at meals in order not to gain weight. (CR)

Page break

1. I do not eat some foods because they make me fat. (CR)
2. I am always hungry enough to eat at any time. (UE)^2^
3. How often do you feel hungry? (UE)^D^ **
4. How frequently do you avoid `stocking up' on tempting foods? (CR)^D^
5. How likely are you to consciously eat less than you want? (CR)^D^
6. Do you go on eating binges though you are not hungry? (UE)^2^ ***
7. On a scale of 1 to 8, where 1 means no restraint in eating (eating whatever you want, whenever you want it) and 8 means total restraint (constantly limiting food intake and never `giving in'), what number would you give yourself? (CR)

---

* The letters in brackets after item indicate the factor in TFEQ-R18 version.

** Finnish version states: “How often do you fancy/desire food?” (English translation by the author)

*** Finnish version states: “Do you continue generous eating, even you are not hungry?” (English translation by the author)

The sub item of UE has been indicated with number ^1^ or ^2^ after brackets depending on whether the item belongs to UE1 or UE2 subfactor.

The three deleted items are indicated with ^D^ after the brackets.

Page break means that the paper version the questionnaire continues at the other side of the paper.

Scale of items from 1 to 13 is: definitely true / mostly true / mostly false / definitely false

Scale of item 14 is: only at mealtimes / sometimes between meals / often between meals / almost always

Scale of item 15 is: almost never / seldom / usually / almost always

Scale of item 16 is: unlikely / slightly likely / moderately likely / very likely

Scale of item 17 is: never / rarely / sometimes / at least once a week

Scale of item 18 is: eat whatever I want, whenever I want it / constantly limiting food intake, never `giving in'
