# Supplementary material for: Psychometric evaluation of three-factor eating questionnaire -R18 in aging Finnish men with increased risk for type 2 diabetes
Source: Nutr Health. 2022 Jul 11;30(2):279–90. doi: 10.1177/02601060221112178 (PMC11141102; doi:10.1177/02601060221112178)
Supplement: sj-docx-2-nah-10.1177_02601060221112178 - Supplemental material for Psychometric evaluation of three-factor eating questionnaire -R18 in aging Finnish men with increased risk for type 2 diabetes [file sj-docx-2-nah-10.1177_02601060221112178.docx]

Psychometric evaluation of Three-Factor Eating Questionnaire -R18 in ageing Finnish men with increased risk for type 2 diabetes

Supplementary table S2. Correlations of factors (Spearman’s rho).

|  | | **CR**  **6i** | **CR w/o i16** | **CR**  **4i** | **UE**  **9i** | **UE1 Cravings** | **UE2 LoC** | **UE**  **8i** |
| --- | --- | --- | --- | --- | --- | --- | --- | --- |
| **CR w/o i16** | r | 0.967*** |  |  |  |  |  |  |
| **CR 4i** | r | 0.932*** | 0.950*** |  |  |  |  |  |
| **UE 9i** | r | -0.151** | -0.106† | -0.105† |  |  |  |  |
| **UE1 Cravings** | r | -0.049 | -0.016 | -0.016 | 0.780*** |  |  |  |
| **UE2 Loss-of-control** | r | -0.163*** | -0.114† | -0.117† | 0.927*** | 0.533*** |  |  |
| **UE 8i** | r | -0.127** | -0.08 | -0.08 | 0.980*** | 0.861*** | 0.881*** |  |
| **EE 3i** | r | 0.047 | 0.096† | 0.079 | 0.537*** | 0.519*** | 0.447*** | 0.550*** |

**CR**: Cognitive Restraint, **UE**: Uncontrolled Eating, **EE**: Emotional Eating, **CR w/o i16**: Cognitive Restraint factor without item 16, **CR 4i**: Cognitive Restraint factor with 4 items, **UE1 Cravings**: subfactor 1 of Uncontrolled Eating, **UE2 LoC**: subfactor 2 of Uncontrolled Eating, **UE high**: higher-order factor of Uncontrolled Eating, **r**: Correlation Coefficient. Significance of Correlations: † p < 0.100, ** p < 0.010, *** p < 0.001.
